# Porosity of Calcium Silicate Hydrates Synthesized from Natural Rocks

**DOI:** 10.3390/ma14195592

**Published:** 2021-09-26

**Authors:** Raimundas Siauciunas, Giedrius Smalakys, Tadas Dambrauskas

**Affiliations:** Department of Silicate Technology, Kaunas University of Technology, Radvilenu pl. 19, 50270 Kaunas, Lithuania; giedrius.smalakys@gmail.com (G.S.); tadas.dambrauskas@ktu.lt (T.D.)

**Keywords:** tobermorite, xonotlite, porosity, BET analysis, insulating materials

## Abstract

In this work, the suitability of natural raw materials with various modifications of SiO_2_—granite sawing waste (quartz) and opoka (a mixture of cristobalite, tridymite, quartz, and an amorphous part)—for the 1.13 nm tobermorite and xonotlite synthesis is examined, and their specific surface area, pore diameter and volume, and the predominant pores are determined. Hydrothermal syntheses were carried out at 200 °C for 12 and 72 h from mixtures with a molar ratio of CaO/SiO_2_ = 1.0. X-ray diffraction analysis, simultaneous thermal analysis, and scanning electronic microscopy were used, which showed that in the lime–calcined opoka mixture the formation of crystalline calcium silicate hydrates takes place much faster than in the lime–granite sawing waste mixture. The high reactivity of amorphous SiO_2_ results in the rapid formation of 1.13 nm tobermorite and xonotlite (12 h). According to Brunauer, Emmet and Taller (BET) analysis data, this product features a specific surface area of ~68 m^2^/g, a total pore volume of 245 × 10^−3^ cm^3^/g, and has dominating 1–2.5 nm and 5–20 nm diameter pores. This porosity of the material should provide good thermal insulation properties of the products made from it as no air convection occurs in the fine pores.

## 1. Introduction

The main approach to reducing energy losses is the usage of efficient insulating materials. Organic insulating materials (polyurethane, polystyrene) have excellent technical properties [1,2,3], however they are thermolabile [4]. Furthermore, cellulose, which is one of the most environmentally friendly and fire-resistant types of insulation [5], only works in the temperature range of 50–550 °C and can cause allergic diseases [6]. Thus, when the temperature reaches above 500 °C, silicate and ceramic materials have to be used [7]. For this reason, fiberglass is widely used to produce high-temperature thermal insulation materials [8]. However, the glass fibers can damage the eyes, lungs, and even the skin [9]. Meanwhile, stone wool, which is a great alternative and non-flammable, has to be covered with expensive organic or inorganic binders to be used at temperatures above 400 °C [10,11]. One of the most effective industrial insulation materials in the world is aerogel [12,13], which is resistant to high temperatures (1100 °C) and requires 50–80% less thickness than other insulation materials. However, aerogels are very expensive materials and so far, are only used for niche purposes.

One of the most efficient types of insulating products with an operating temperature of 1050 °C are materials based on calcium silicates and/or calcium silicate hydrates: aerogel–xonotlite composite [14]; ultra-light xonotlite [15]; xonotlite-type calcium silicate [16], and needle-like xonotlite–tobermorite mixture [17], the properties of which are regulated by Standard EN 16977: 2020 [18]. Calcium silicate hydrates can be produced by using widespread materials: limestone, quartz sand, silica fume [19]; as well as fibers, biomass ashes [20] and other unconventional materials [21]. The most important performance characteristics of calcium silicates and calcium silicate hydrates are low density and thermal conductivity, high mechanical strength [22], good heat resistance, low shrinkage up to 1050 °C, durability and resistance to chemical corrosion [23], and large specific surface area and nano-scale particles size, which is why they can be applied in catalysis of hydrogen generation [24]. The thermal stability of calcium silicate hydrates (xonotlite, tobermorite, and others) depends on their recrystallization to wollastonite (usually 800–950 °C) [25], as well as impurities that change their shrinkage during calcination. To improve thermal stability, it is necessary to synthesize pure calcium silicate hydrates [26], i.e., reduce the amount of amorphous and/or semi-crystalline phases that result in high shrinkage values.

Xonotlite, Ca_6_Si_6_O_17_(OH)_2_, one of the most important calcium silicate hydrates, hardly shrinks during thermal transformation because its structure is very similar to wollastonite [27]. In addition, the latter compound has the lowest content of crystalline water, is the most heat-resistant, and is the most thermally stable (decomposition temperature 1050–1100 ℃) of all calcium silicate hydrates. Thus, xonotlite is widely used not only for the production of insulation and refractory materials, ceiling and wallboards, architectural and lightweight panels, but for the other applications too, as a catalyst [28], as polymers [29], and in medical applications [30]. Hydrothermal synthesis of this compound goes in the most favorable way when the molar ratio of the initial mixture CaO/SiO_2_ is equal to 1.0 while the temperature of curing is 200–220 °C [31].

Another calcium silicate hydrate produced industrially is 1.13 nm tobermorite, Ca_5_Si_6_O_16_(OH)_2_·4H_2_O. This compound forms in autoclaved aerated concrete, sand-lime bricks, during the hydration of ordinary Portland cement (OPC), and in heat insulating materials. In addition, the unique morphology of 1.13 nm tobermorite crystals (the solid state, the fibers and nanoparticles) allows it to be used for the sorption of heavy metals ions and nuclear wastes in the slurry, for the production of semiconductors, and in medicine [32]. This calcium silicate hydrate remains stable in the mixtures within the range of the molar ratio CaO/SiO_2_ = 0.66–0.83 and the temperature of hydrothermal treatment 180–200 °C [33]. The properties of heat-resistant products based on 1.13 nm tobermorite are very close to those of xonotlite-based products, but the application temperature is much lower, 650–700 °C.

The mechanism of xonotlite and 1.13 nm tobermorite formation is a complex process and highly dependent on the reactivity of the raw materials since various intermediate compounds can be formed during synthesis. The synthesis of calcium silicate hydrates is also significantly influenced by hydrothermal treatment conditions (temperature, duration, intensity of stirring, etc.). Thus, the properties of the raw materials and the autoclave mode are the main parameters that need to be controlled or changed. To successfully apply natural raw materials for the synthesis of calcium silicate hydrates it is necessary to carry out systematic studies of hydrothermal curing parameters, because the changes of one of the conditions can unpredictably affect the whole process of compound formation and properties.

It is worth mentioning that the thermal conductivity of thermal insulating heat-resistant products is determined not so much by the mineral composition of the solid phase as by the specific surface area of the material, the total volume of the pores, their shape and radial distribution. In the case of products based on calcium silicate hydrates, these issues have so far been addressed only in fragments in the scientific literature.

The aims of this work are to compare the suitability of natural materials with various modifications of SiO_2_ (quartz; a mixture of cristobalite, tridymite, quartz and an amorphous part) for the economically attractive xonotlite and 1.13 nm tobermorite synthesis, to determinate their specific surface area, pore diameter and volume, the model of predominant pores, and to assess whether they meet the requirements for thermal insulating materials.

## 2. Materials and Methods

### 2.1. Characterization of the Raw Materials

In this work the following materials were used:

- Silica-calcite sedimentary rock opoka (Stoniskis-Zemaitkiemis quarry, Silute, Lithuania). The chemical and mineralogical composition of opoka is given in Table 1 and Table 2, respectively. It was obtained that opoka mainly consists of four different modifications of SiO_2_: quartz, cristobalite, and tridymite (in total 35 wt%) and amorphous SiO_2_ (19.6 wt%). Since opoka contains calcite, it was calcined at 775 °C for 1 h and later milled in a ball mill to reach Sa ≈ 970 m^2^/kg [34]. The amount of CaO_free_ from total CaO was equal to 50.67%. It was obtained that crystalline modifications of SiO_2_ remained stable during calcination, while the amorphous part of SiO_2_ reacted with calcium oxide and as a result wollastonite and larnite were formed.
- Granite sawing powder waste (GSW) (JSC Granitas, Kaunas, Lithuania) was milled in a ball mill until Sa ≈ 905 m^2^/kg. The chemical and mineral compositions of granite sawing powder waste are given in Table 1 and Table 2, respectively.
- Limestone (Karpenai quarry, Naujoji Akmene, Lithuania) consists of 93.42% calcite, 3.23% dolomite, 2.61% quartz and 0.74% other impurities. It was additionally calcined at 900 °C for 1 h in a laboratory kiln Nabertherm LV 15/11/P330 (Lilienthal, Germany) and later milled until S_a_ ≈ 650 m^2^/kg. CaO_free_ = 91.2%.

More detailed characterization (X-ray diffraction analysis, differential scanning calorimetry, thermogravimetry, granulometry) of the raw materials has been published in detail in our previous work [35].

### 2.2. Experimental Methods

#### 2.2.1. Hydrothermal Synthesis

For the synthesis of calcium silicate hydrates two different mixtures were performed: (1) lime—calcined opoka; (2) lime—granite sawing powder waste. The molar ratio of CaO/SiO_2_ was equal to 1.0. The weighted materials were mixing in a homogenizer Turbula Type T2F (Muttenz, Switzerland) for 1 h at 49 rpm.

Hydrothermal synthesis was carried out in unstirred suspensions (W/S = 10.0) in 25 mL volume PTFE cells, which were placed in “Parr Instruments” autoclave under saturated steam pressure at 200 °C; the duration of isothermal curing was 12 or 72 h. The products were filtered, rinsed with acetone to reduce carbonization, dried at a 100 ± 1 °C temperature, and put through a sieve with an 80-μm mesh.

#### 2.2.2. Instrumental Analysis

The surface area of the raw materials was determined using laser particle size analyzer CILAS 1090 LD (Cilas, Orleans, France) with a sensitivity range from 0.04 µm to 500 µm.

The chemical composition analysis of samples was performed by X-ray fluorescence spectroscopy (XRF) on a Bruker X-ray S8 Tiger WD spectrometer (Bruker AXS GmbH, Karlsruhe, Germany) equipped with Rh tube with energy of up to 60 keV. Powder samples were measured in Helium atmosphere and data were analyzed with SPECTRA Plus QUANT EXPRESS standard less software (Bruker AXS GmbH, Karlsruhe, Germany).

The X-ray diffraction analysis (XRD) was performed on the D8 Advance diffractometer (Bruker AXS GmbH, Karlsruhe, Germany) operating at the tube voltage of 40 kV and tube current of 40 mA. The X-ray beam was filtered with Ni 0.02 mm filter to remove the CuK_β_ wavelength. Diffraction patterns were recorded in a Bragg–Brentano geometry using a fast-counting detector Bruker Lynx Eye (Bruker AXS GmbH, Karlsruhe, Germany) based on a silicon strip technology. The samples were scanned over the range 2*θ* = 3–70° at a scanning speed of 6° min^−1^ using a coupled two theta/theta scan type. The software Diffrac.eva v3.0 Bruker AXS GmbH, Karlsruhe, Germany) was used for compounds identification.

XRD analysis was complimented by Rietveld refinement. For this, 10% of ZnO was added to sample as an internal standard for the quantitative determination of the amorphous phase. The samples for the quantitative X-ray diffraction analysis (QXRD) analysis were dried at 40 °C for 48 h and ground to pass a 32 μm sieve. Refinement was performed using Diffrac.Topas 4.2 software (Bruker AXS GmbH, Karlsruhe, Germany). This method was used to determine the quantitative mineral composition of raw materials.

XRD software Diffrac.Eva was used for the calculation of crystallite size. The crystallite size of 1.13 nm tobermorite from crystalline plane (*h k l*; *d*–spacing 1.133 nm) and xonotlite (*h k l*; *d*–spacing 0.702 nm) was determined following the Scherrer equation:

(1) $$D_{hkl}=\frac{k_{hkl}\cdot \lambda }{\beta_{hkl}\cdot \cos\theta }$$

where *λ* is the wavelength of the Cu K_α_ radiation, *θ* is Bragg’s diffraction angle, *β_hkl_* is the full width at half maximum intensity, and *k* is a shape factor (the value used in this study was 0.94).

The peak area and intensity computations were performed on an interval between two points, called “entry points” or Left Angle and Right Angle. These are the angles (in degrees) of the scan point that are the closest from the entry points. These are statistical computations assuming there is a unique peak in the interval. It supplies information about the position of the peak maximum and the net area of the peak. Dedicated software Diffrac.Eva was used for this purpose. It is worth mentioning, that the instrumental broadening was not considered.

The highest value in the interval may not be pertinent information due the noise fluctuations. The position of the peak maximum was located by fitting a parabola through the points around the highest value and is given in scan unit (plus *d* in Å, because the scan was 2*θ*). The output Gross height is the intensity of the fitting parabola, in cps. We calculated the Net height, which is obtained from the Gross height minus the background intensity, which is determined by a linear background between the left and right extremities. Software Diffrac.Eva was used for this purpose.

Thermal analyzer Linses PT1000 was applied to simultaneous thermal analysis (STA; differential scanning calorimetry DSC, and thermogravimetry TG) studies. The heating was carried out under N_2_ atmosphere at a heating rate of 10 °C·min^−1^; the temperature ranged from 40 up to 1000 °C. The ceramic sample handlers and crucibles of Pt were used.

The microstructure of the materials was observed using scanning electron microscopy (SEM) (Model JSM-7600F, JEOL Co., Tokyo, Japan) coupled with energy dispersive X-ray spectrometry (EDX) (Inca Energy 350, Silicon Drift type detector X-Max20, Oxford Instruments, Abingdon, UK) performed by using accelerated voltage of 10 kV, the working distance of 8.6 and 8.7 mm for SEM observation, and 200 s accumulation time for EDX analysis. The powder samples were sputter coated with gold to promote electrical conductivity.

#### 2.2.3. Determination of Pore Characteristics

The specific surface area of the synthesis products was determined by Brunauer, Emmet and Taller (BET) method. Measurements were performed on a KELVIN 1042 Sorptometer (Costech Instruments, Via Firenze, Italy). The specific surface area was calculated according to the BET equation using N_2_ adsorption isotherm data in the range of (0.05 < *p*/*p*_0_ < 0.35) (Equations (2)–(8) were taken from [36]):

(2) $$\frac{1}{X\left(\frac{p_{0}}{p}-1\right)}=\frac{C-1}{X_{m}\cdot C}\cdot \frac{p}{p_{0}}+\frac{1}{X_{m}\cdot C},$$

where *X* is the mass adsorbed on the sample at relative pressure *p*/*p*_0_ (*p* is the partial pressure of adsorbate and *p*_0_ is the saturated vapor pressure of adsorbate), *X_m_* is the mass of N_2_ adsorbed on the surface, when monolayer is formed, *C* is a constant which is a function of the heat of the adsorbate condensation and the heat of adsorption (C_BET_).

The values of BET equation are correct if yields are in a straight line when $\frac{1}{X\left(\frac{P_{0}}{p}-1\right)}$ is plotted $\frac{p}{p_{0}}$. The angle of slope $S=tg\alpha =\frac{C-1}{X_{m}\cdot C}$ and the length of the segment intersected in the ordinate axis $I=\frac{1}{X_{m}\cdot C}$ were used to determine *X_m_* and *C*. In order to calculate these parameters, the equations was rewritten as follows: $X_{m}=\frac{1}{S+I}$ and $C=\frac{1}{I\cdot X_{m}}$. The BET equation plot provides linear change in the range of *p*/*p*_0_ = 0.05–0.35. The total surface area *S_t_* of the sample is calculated from the equation:

(3) $$S_{t}=\frac{X_{m}\cdot N\cdot A_{cs}}{M}$$

where *A_cs_* is the cross-sectional area of the adsorbate molecule (*A_ad_* = 16.2⋅10^−20^ m^2^), *M* is molecular weight of adsorbate, and *N* is Avogadro constant (6.023⋅l0^23^). The specific surface area is calculated from the equation:

(4) $$S_{BET}=\frac{S_{t}}{m}$$

where *m* is the mass of the sample of product obtained after hydrothermal synthesis.

The total pore volume and the radial distribution of the pores were calculated according to the correlated Kelvin equation and Orr et al. scheme using the N_2_ desorption isotherm at 77 K. The Kelvin equation describes the vapor pressure depression of an adsorbate in a capillary of a given radius:

(5) $$ln\frac{p}{p_{0}}=-2\frac{\gamma \cdot V_{m}\cdot \cos\theta }{R\cdot T\cdot r_{K}}$$

where *p* is the saturated vapor pressure in equilibrium with the adsorbate condensed in a capillary or pore, *p*_0_ the normal adsorbate saturated vapor pressure, *γ* is the surface tension of nitrogen at its boiling point (*γ* = 8.85 erg·(cm^2^)^−1^), *V_m_* is the molar volume of liquid adsorbent N_2_ (*V_m_* = 34.7 cm^3^·mol^−1^), *θ* is surface adsorption angle (usually taken 0° and cos*θ* = 1), *R* is universal gas constant (*R* = 8.134⋅10^7^ ergs·deg^−1^·mol^−1^), T the boiling point of liquid nitrogen (*T* = 77 K) and *r_K_* is the Kelvin radius of pore.

The thickness t of the adsorbed layer is calculated according to this equation:

(6) $$t=\frac{V_{a}}{V_{m}}\cdot \tau ,$$

where *V_m_* is volume of absorbed gas, mm^3^/g and *τ* is thickness of monomolecular adsorbent, mm.

Since we use a cylindrical pore model $r_{p}=r_{k}+t$ then we get the equation:

(7) $$V_{p}={\left(\frac{{\bar{r}}_{p}}{{\bar{r}}_{k}}\right)}^{2}\cdot \left(V_{L}-\Delta t\cdot \sum A\right),$$

where ${\bar{r}}_{p}$ is the actual average of pore radius in the range *r*_2_ − *r*_1_, Ä.

The volume of desorbed liquid in any desorption isotherm range is related to the volume of gas evolved Δ*V_p_* = Δ*V_d_* (1.54 × 10^−3^). Cylindrical pore is calculated by the equation:

(8) $$A=\frac{2\cdot \Delta V_{p}}{{\bar{r}}_{p}}\cdot 10^{4}$$

where Δ*V_p_* is volume of gas emitted, cm^3^.

Parallel plate type pairs are calculated using the following Equations (9)–(11) [37]:

(9) $$\bar{d_{p}}=r_{k}+2t,$$

where *r_k_* is the measured and $\bar{d_{p}}$ the actual distance between two plates, Ä.

The average pore distance between two plates $\bar{d_{p}}$ volume *V_p_* is equal:

(10) $$V_{p}=\frac{\bar{d_{p}}}{{\bar{r}}_{k}}\left(\Delta V_{L}-2\Delta t\sum^{\;}A\right)$$

The theoretical surface area *A* of the pore walls is calculated by this equation:

(11) $$A=\frac{2V_{p}}{d_{p}}$$

The calculations are completed using any model when Δ*t*·Σ*A* exceeds Δ*V_L_* value.

## 3. Results

### 3.1. Characterization of Synthesis Products

**Figure 1:**
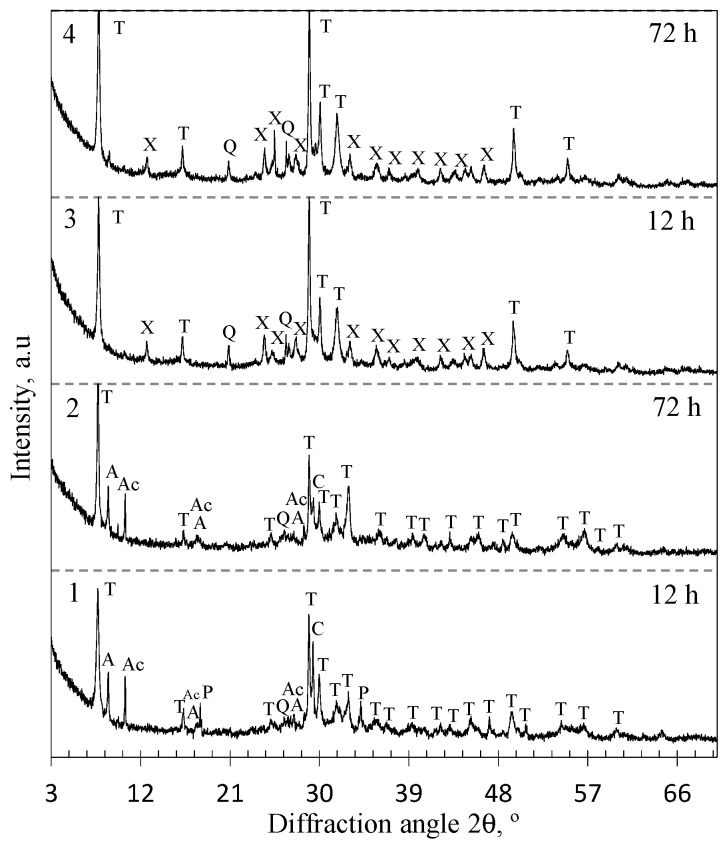
XRD patterns of the synthesis products from lime–GSW (patterns 1, 2) and lime–calcined opoka (patterns 3, 4) mixtures with CaO/SiO_2_ = 1.0 at 200 °C. Indexes: A—annite, Ac—actinolite, C—calcite, P—portlandite, Q—quartz, T—1.13 tobermorite, X—xonotlite.

It was determined, that in the lime–GSW mixture, after 12 h of isothermal curing at 200 °C when CaO/SiO_2_ = 1.0 (unstirred suspensions), only one crystalline compound 1.13 nm tobermorite (*d* = 1.133; 0.548; 0.308; 0.298; 0.282; 0.184 nm) was formed (Figure 1, pattern 1). A possible reason why lower basicity of 1.13 nm tobermorite (CaO/SiO_2_ = 0.83) with a high degree of crystallinity is formed already at the beginning of the synthesis is that the GSW contains Na^+^, K^+^, and Al^3+^ ions-containing minerals (albite NaAlSi_3_O_8_, anorthite CaAl_2_Si_2_O_8_, and labradorite Ca_0.52_Na_0.48_(Si,Al)_4_O_8_), which promote the formation of 1.13 nm tobermorite. It is worth mentioning that annite KFe_3_AlSi_3_O_10_(OH)_2_ (*d* = 1.01; 0.337; 0.266; 0.218; 0.118 nm) and actinolite K_0.01_Na_0.05_Ca_1.9_Mg_3.4_Mn_0.1_Fe_1.5_Al_0.2_Si_7.9_O_22.1_(OH)_1.9_ (*d* = 0.906; 0.845; 0.489; 0.313; 0.244 nm) do not participate in the formation reactions of calcium silicate hydrates because the intensive diffraction peaks characteristic to these compounds were identified (Figure 1, pattern 1). This can be explained by the fact that the solubility of these minerals in water is very low and Ca^2+^ ions could not react with them. Traces of unreacted quartz (*d* = 0.426; 0.335; 0.228; 0.182 nm) and portlandite (*d* = 0.425; 0.335; 0.245 nm) are also found, which indicates that the hydrothermal reactions are not over yet. It was determined that synthesis products partially carbonized during the drying of the product because of low intensity peaks of calcite (*d* = 0.303; 0.228; 0.192 nm) were identified.

**Figure 2:**
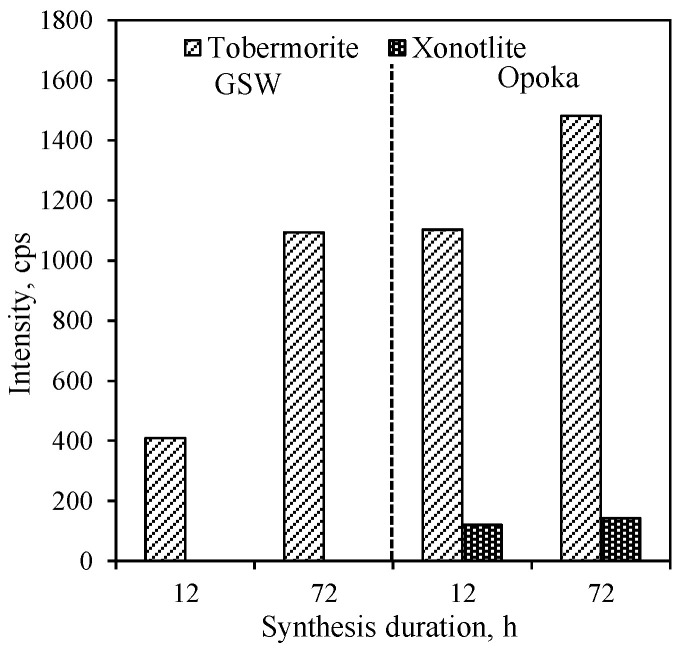
Intensity of the tobermorite (*d* = 1.13 nm) and xonotlite (*d* = 0.702 nm) peaks obtained from lime–GSW and lime–calcined opoka mixtures.

**Figure 3:**
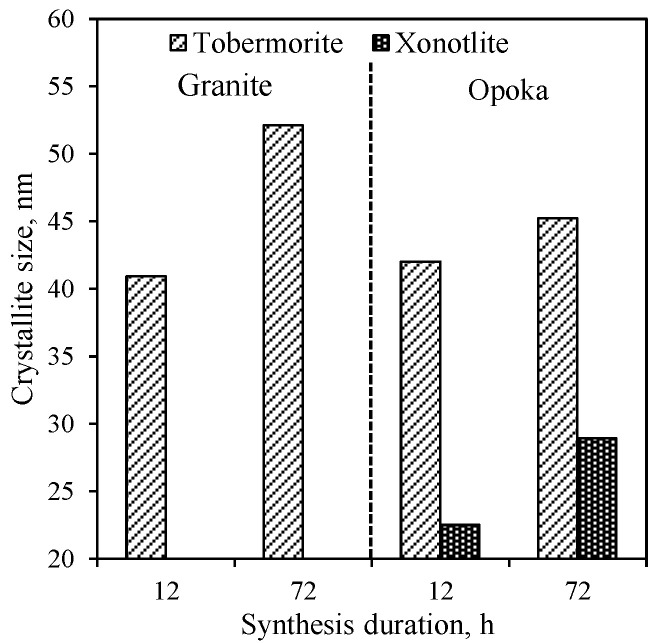
Dependence of the 1.13 nm tobermorite and xonotlite crystallite size on the duration of synthesis from lime–GSW and lime–calcined opoka mixtures.

By extending the synthesis duration to 72 h, the intensity of peaks characteristic to the main synthesis product 1.13 nm tobermorite increases significantly (Figure 1, curve 2), while the intensity of the main peak (*d* = 1.13 nm) increases in value from 409 to 1094 cps (Figure 2). In addition, under these synthesis conditions crystallite size (determined by Scherrer method) of 1.13 nm increases from 40.9 nm (after 12 h of curing) to 52.1 nm (after 72 h of curing), which is almost three times higher (Figure 3). However, a huge amount of Al^3+^ ions presented in raw mixture prevents the formation of xonotlite, despite the fact that the composition of the mixture corresponds to its stoichiometry, and the synthesis temperature is optimal for the crystallization of this compound. A similar phenomenon has been observed previously in the synthesis of gyrolite and other calcium silicate hydrates [38]. In addition, the annite and actinolite presented in the GSW do not react and remain in the synthesis products even after 72 h of isothermal current.

In order to determine the influence of natural raw materials with various modifications of SiO_2_ (quartz in GSW; a mixture of cristobalite, tridymite, quartz and an amorphous part in opoka) on the formation of calcium silicate hydrates, the hydrothermal synthesis at 200 °C from a lime–opoka mixture with a molar ratio of CaO/SiO_2_ = 1.0 was performed as well. It was determined that after 12 h of isothermal curing, 1.13 nm tobermorite was identified as the main compound in the product (Figure 1, pattern 3). In contrast to the mixtures with GSW, xonotlite also forms. In addition, quartz did not react fully while other SiO_2_ compounds (tridymite and cristobalite and amorphous part) as well as portlandite were fully reacted. Furthermore, XRD analysis data shows that the mineralogical composition of synthesis products did not change by prolonging the duration of the hydrothermal treatment to 72 h (Figure 1, patterns 3 and 4). It was obtained that the intensity of main diffraction peak characteristic of the xonotlite increases from 120 cps to 142 cps while the 1.13 nm tobermorite peak (*d*—1.13 nm) increases from 1103 cps to 1481 cps (Figure 2). The size of the crystallites of the formed compounds changes are completely different. In the case of 1.13 nm tobermorite this value increases only slightly (from 42 to 45.2 nm), while in the case of xonotlite increases by almost 30% (from 22.5 to 28.9 nm) (Figure 3). Moreover, when comparing with the products obtained in the mixture of lime–GSW, it was determined that, after 72 h of isothermal curing, the highest intensity of 1.13 nm tobermorite was obtained when using lime–opoka mixtures. It can be assumed that synthesis goes in a more favorable direction when in the mixtures there is some quantity of amorphous SiO_2_ and impurities, which accelerates the formation of calcium silicate hydrates.

**Figure 4:**
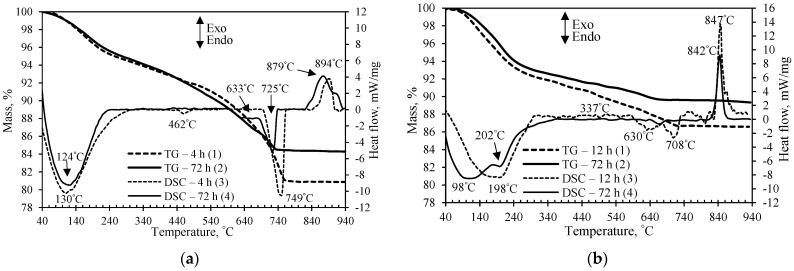
TG (1, 2) and DSC (3, 4) curves of the synthesis products from lime–GSW (**a**) and lime–calcined opoka (**b**) mixtures with CaO/SiO_2_ = 1.0 at 200 °C after 12 h (1, 3) and 72 h (2, 4).

The results of simultaneous thermal analysis confirmed and supplemented the XRD data. The STA curves of the synthesis products obtained in the mixtures of lime and GSW are given in Figure 4a. The first endothermal effect at temperature of 40–240 °C shows the dehydration of 1.13 nm tobermorite together with semi-amorphous C-S-H type calcium silicate hydrates. However, this effect is very broad and vaguely expressed; due to this reason, it is impossible to distinguish C-S-H dehydration from 1.13 nm tobermorite (Figure 4a, curves 3 and 4). Other authors obtained similar results during the synthesis of 1.13 nm tobermorite from natural rocks and industrial waste materials with Al-containing impurities [26,39]. The second endothermal effect at 462 °C can be assigned to the decomposition of portlandite, which confirms that portlandite did not fully reacted after 12 h of synthesis (Figure 4a, curve 3).

In both synthesis products, the mass loss in the 180–640 °C range is about 9.5% (Figure 4a, curves 1 and 2). The 1.13 nm tobermorite loses most of its crystalline water (4.5 molecules) before reaching 240 °C, and the remaining 0.5 molecules show this effect only at ~700 °C. Meanwhile, semi-amorphous C-S-H loses water slowly but consistently over the entire mentioned temperature range. Thus, the TG results show that the products synthesized from lime–GSW mixture at 200 °C contain a sufficient amount of semi-amorphous C-S-H type calcium silicate hydrates.

The endothermic effect at 725–750 °C shows the decomposition of carbonates, and the mass loss of the treated product for 12 h in the temperature range of 707–761 °C is fairly large, at 5.07% (Figure 4a, curve 1). However, in the synthesis product obtained after 72 h, a significant decrease of carbonates was observed because the mass loss is almost three times smaller and equal to 1.73%. This indirectly indicates that far fewer semi-amorphous compounds remain in the synthesis product because they carbonize much more readily than crystalline calcium silicate hydrates [40]. This is in good agreement with the literature data which indicates that some quantity of synthesis products could be carbonated when the samples are prepared for instrumental analysis [41,42].

The exothermal effect at 894 °C was assigned to semi-amorphous C-S-H recrystallization to wollastonite (Figure 4a, curve 3). Moreover, the exothermic effect at 879 °C in the synthesis product obtained after 72 h of hydrothermal treatment remains (Figure 4a, curve 4). It is known that the higher the basicity of semi-amorphous C-S-H, the higher is its recrystallization temperature to wollastonite [25]. Thus, it can be stated that the 12-h synthesis product contains C-S-H(II) (with exothermic effect at 894 °C), and the 72-h synthesis product contains C-S-H(I) and C-S-H(II) (at 879 °C and 902 °C) type calcium silicate hydrates. Most likely, during 72 h of synthesis, almost all of the quartz dissolves and the molar ratio of CaO/SiO_2_ in the reacting medium approaches the composition of the initial mixture (CaO/SiO_2_ = 1.00).

It was determined that samples synthesized in the lime–calcined opoka mixture are characterized by well-expressed endothermic effect at 80–240 °C temperature (Figure 4b). This effect in the product synthesized for 72 h is divided into two stages, and dehydration of 1.13 nm tobermorite is clearly visible. The negligible endothermal effect at 337 °C shows that the hydrogarnets (3CaO·Al_2_O_3_(3 − *x*)SiO_2_·2*x*H_2_O, where *x* may vary from 0 to 3) formed at the beginning of the synthesis (Figure 4b, curve 3). Thus, it can be assumed that still not all aluminum ions were intercalated into the 1.13 nm tobermorite crystal lattice. The endothermic effect of the decomposition of carbonates is negligible, which confirms that xonotlite and 1.13 nm tobermorite are more resistant to the carbonization than semi-amorphous C-S-H. In addition, TG analysis showed that the total mass loss after 72 h is less than after 12 h of synthesis, and it reached 10.4% (Figure 4b, curve 2). This value is much lower than that of the analogous product from the lime–GSW mixture (14.3%). Probably, this is due to the formation of calcium silicates hydrates of higher crystallinity, in particular xonotlite, which contains only one molecule of water.

**Figure 5:**
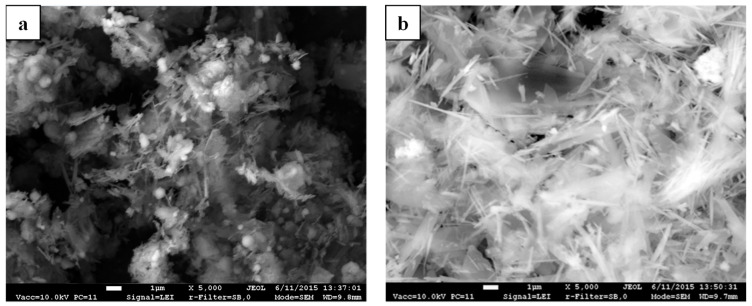
SEM micrograph of synthesis products from lime–GSW (**a**) and lime–opoka (**b**) mixtures with CaO/SiO_2_ = 1.0 after 72 h at 200 °C.

The accumulation of two morphologies of 1.13 nm tobermorite crystals can be noticed in the SEM image of the 72-h synthesis product from the lime–GSW mixture (Figure 5a): 1–2 µm length needle-shaped crystals and 2–4 µm size plates or sheets are visible. This data is very similar to the results presented by other authors [19,43] who noted that 1.13 nm tobermorite crystals can be of two types, shaped either like needles or plates. It can be concluded that, after 72 h of hydrothermal synthesis at 200 °C, 1.13 nm tobermorite is a predominant compound in the synthesis product, and its crystals are distributed over the entire volume of the sample. It should be noted that together with the 1.13 nm tobermorite crystals a small amount of agglomerates characteristic of the semi-amorphous C-S-H type calcium silicate hydrates also is visible.

According to the SEM data, in the 72-h synthesis product from lime–calcined opoka mixture, the needle-shaped crystals of xonotlite were well expressed, however, with 1.13 nm tobermorite crystals in the mix (Figure 5b). It is in good agreement with the XRD analysis data where diffraction peaks characteristic of xonotlite and 1.13 nm tobermorite were identified. Furthermore, the XRD data showed, and SEM analysis confirmed, that the highest crystallinity degree of xonotlite and 1.13 nm tobermorite crystals was obtained after 72 h of isothermal curing. It should also be mentioned that fewer agglomerates of semi-amorphous compounds are visible, and their dimensions are smaller.

### 3.2. Specific Surface Area and Porosity of Synthesis Products

**Figure 6:**
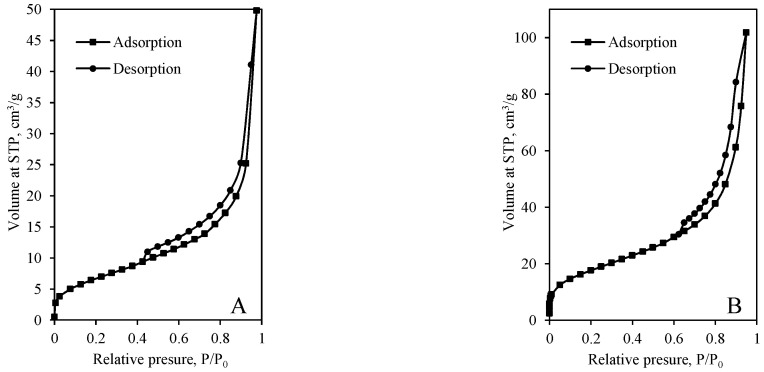
N_2_ adsorption–desorption isotherms of products (CaO/SiO_2_ = 1.0 at 200 °C) after 12-h synthesis from mixtures: lime–GSW (**A**), lime–calcined opoka (**B**).

**Figure 7:**
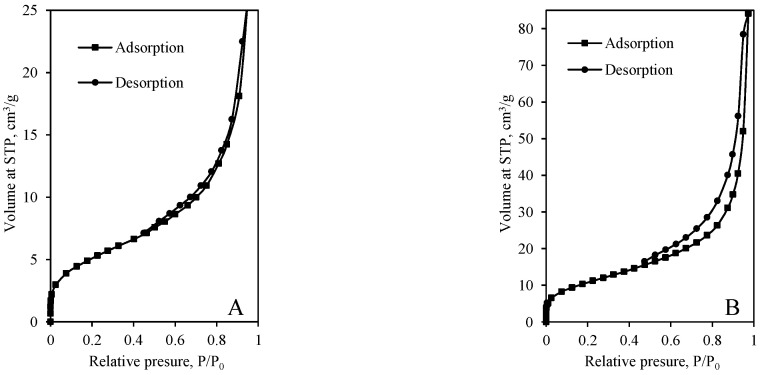
N_2_ adsorption–desorption isotherms of products (CaO/SiO_2_ = 1.0 at 200 °C) after 72-h synthesis from mixtures: lime–GSW (**A**), lime–calcined opoka (**B**).

N_2_ adsorption–desorption isotherms of the samples obtained from various mixtures after 12 h and 72 h of hydrothermal synthesis are presented in Figure 6 and Figure 7. It was determined that the shape of pores depends on both the chemical composition of the primary mixture and the duration of synthesis. The shape of adsorption isotherms of samples synthesized for 12 h can be classified as Type II (the absence of a plateau at higher *p*/*p*_0_ values) or Type IV(a) (the occurrence of the hysteresis loop) (Figure 6). Type II isotherm is characteristic of nonporous or macroporous materials, while Type IV(a) is characteristic of mesoporous adsorbents. Thus, synthesis products are mesoporous materials, although macroporous pores are also possible. Additionally, the sharp increase of isotherms at *p*/*p*_0_ ~0.05 shows that some micropores can be presented in the structure of the synthesis products compounds. It is worth mentioning that the same tendency can be observed in most calcium silicate hydrates [37].

It was determined that all N_2_ adsorption–desorption isotherms of the synthesis products synthesized for 12 h do not coincide, i.e., desorption isotherm is shifted to the left of the adsorption isotherm by forming a hysteresis loop (Figure 6). According to the International Union of Pure and Applied Chemistry (IUPAC) classification, five types of hysteresis loops are distinguished according to the different exhibited forms. It was determined that desorption isotherm of the synthesis product obtained in the mixture of GSW and lime (12 h) is narrow, and it ends at ~0.45 (Figure 6A). Thus, it is difficult to classify the hysteresis loop, and it may be between H1 and H3. Most likely, both types of pores, the cylindrical pores and slit-shaped pores are presented in the sample; thus, the hysteresis loop does not precisely correspond to the classic shape of the hysteresis loop.

Furthermore, the hysteresis loop of the sample obtained in the mixture of lime–calcined opoka (12 h) can be classified as Type H1 because it closes and merges with the absorption curve at fairly high pressure (*p*/*p*_0_ ~0.6) (Figure 6B).

It was determined that synthesis duration strongly affects the shape of dominant pores because the sample synthesized in GSW and lime mixture for 72 h becomes nonporous because adsorption isotherm is reversible, i.e., coincides with desorption isotherm (Figure 6A and Figure 7A). Such type of isotherm is classified as Type II and is typical for nonporous materials. In this case, the Kelvin equation cannot be used to calculate the dominant pore parameters.

Further analysis showed that the microstructure of the sample obtained by using lime–calcined opoka also changed significantly. It was determined that the value of relative pressure when desorption isotherm merges with the absorption isotherm decreases from ~0.6 to ~0.45 (Figure 6B and Figure 7B). The shape of the hysteresis loop is similar to H3, which is characteristic to slit-shaped pores. Most likely, the prolonged synthesis duration leads to a change of the pore shape from cylindrical shape into slit shape.

**Figure 8:**
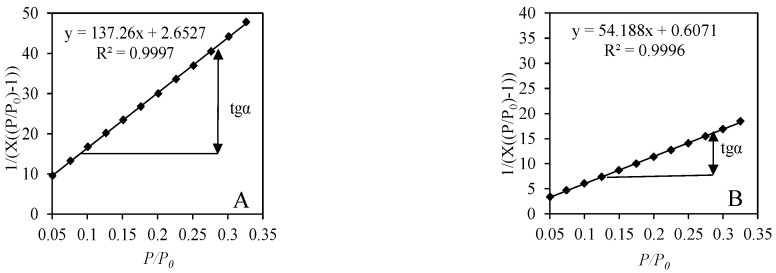
Isotherm of N_2_ adsorption at 77 K in BET plot of products after 12-h synthesis from mixtures: lime–GSW (**A**), lime–calcined opoka (**B**).

**Figure 9:**
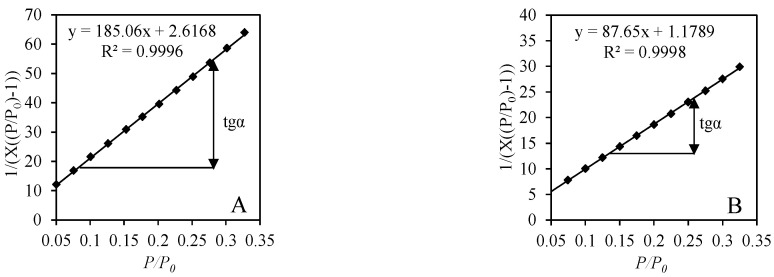
Isotherm of N_2_ adsorption at 77 K in BET plot of products after 72-h synthesis from mixtures: lime–GSW (**A**), lime–calcined opoka (**B**).

Since all adsorption isotherms were classified as Type II or Type IV BET equation can be used to calculate the specific surface area of synthetic samples. For the calculations, the collected data of N_2_ adsorption at the range of relative pressure 0.05 ≤ *p*/*p*_0_ ≤ 0.35 was used. It was determined that, for all the samples, the BET equation yielding a linear plot was obtained in the BET coordination (1/(*X*[(*p*/*p*_0_) − 1)) vs. (*p*/*p*_0_) (Figure 8 and Figure 9). In all cases, the correlation coefficient R^2^ remains very close to the unit, i.e., >0.99.

The most reliable results of *S_BET_* calculations are obtained when the value of constant C_BET_ is between 50–250. In a case of a lower value of the constant (C_BET_ > 50), monolayer and multilayer adsorption can overlap; however, errors obtained by using the BET equation are insignificant until the value of the constant C_BET_ is higher than 10. Conversely, C_BET_ > 250 shows that a chemical interaction takes place between the surface of the adsorbent and the adsorbate without the formation of a monolayer. It was determined that in all cases the calculated constant C_BET_ varies within the values of 52.74–87.38, which perfectly fits into the theoretical guidelines (Table 3). These results show that a stable monolayer was formed on the surface of hydrothermally synthesized samples from various raw materials, and, for this reason, it is possible to calculate the value of *S_BET_* accurately.

It was determined that the specific surface area (*S_BET_*) significantly depends on the composition of raw materials and the duration of hydrothermal synthesis (Table 3). The highest value of *S_BET_* is obtained from the samples synthesized by using lime and calcined opoka, while the lowest value is obtained from the samples synthesized by using lime and GSW. In the samples obtained after 72 h of synthesis, the capacity of monolayer *X_m_* decreases significantly. As a result, the value of calculated *S_BET_* is decreased by 38% in the sample obtained from lime–calcined opoka mixture and by 25% from lime–GSW. Such a decrease in the surface area after a longer duration of hydrothermal synthesis is considered as the increased crystallinity degree of the synthesis products.

The results of adsorption isotherms and hysteresis loops classification showed that the determination of pore shape is complicated. Thus, to accurately determine the shape of the dominant pores in the polydisperse system, the corrected Kelvin equation and the scheme as developed by Orr et al. were used. When the correct shape of pores is determined, the total pore volume and the radial distribution of the pore can be calculated. Since hysteresis loops can be classified as Type H1 or Type H3 the calculations were performed by using two models: the cylindrical pore, and the slit-shaped pore formed between parallel planes. The initial calculations are analogous and applicable to both models while using the measured amount of the adsorbed nitrogen volume at different relative pressures. The Kelvin radius of the pore and the thickness of the adsorbed nitrogen (N_2_) layer are calculated from the Kelvin and Halsey equations. These values are used in further calculations when using various pore models. The total specific surface area Σ*A* is calculated by summing the theoretical surface area A of the pore walls as the relative pressure decreases. The calculations are completed when the difference between the total specific surface area and the nitrogen layer thickness (∆*t*·Σ*A*) becomes greater than the value of the change in the volume (∆*V_L_*) of the evaporated liquid adsorbate. This indicates that the desorbed gas is not of the vapor origin of the liquid walls, but simply a desorbed gas. The most suitable pore model is the one of the experimentally measured specific surface area whose *S_BET_* value is the closest to the calculated Σ*A* value. The summarized results of calculations by using different models describing the pore are given in Table 4.

The calculations of the dominant pore shape of the sample obtained from the lime–GSW mixture after 12 h of synthesis showed that both models did not fit perfectly with the obtained data because the difference between the *S_BET_* value and the calculated Σ*A* value is more than 20% (Table 4). Therefore, it can be stated that the system is polydisperse and dominated by pores of different shapes. These data are in good agreement with the unclear shape of the hysteresis loop (Figure 6B). Moreover, by prolonging the duration of the synthesis, the synthesis products become non-porous or macroporous because the hysteresis loop was not identified (Figure 6B). In addition, these results are confirmed by the small surface area obtained in the sample after 72 h.

It was determined that, in the synthesis product obtained from the lime–calcined opoka mixture, the hysteresis loop is assigned to H1 type, and the predominant pore is of the cylindrical shape. Moreover, when the synthesis duration was prolonged, the shape of the hysteresis loop changed to Type H3, which is characteristic of materials with slit-shaped pores. The results of the calculations using the Kelvin equation coincide with the classification of the hysteresis loop. It was estimated that, after 12 h, cylindrical pores predominate in the sample because the difference between the measurements and the calculations is insignificant (7%), while a much larger difference (40%) was obtained by using the slit-shaped pore model (Table 4). The calculations showed that, with the increase of the synthesis duration to 72 h, the errors obtained by using the slit-shaped and the cylindrical pore model are similar. This indicates that the number of cylindrical pores is decreasing more, and more pores develop between the plates. This is an intermediate state, and it is likely that, by prolonging the duration of synthesis, the slit-shaped pores formed between plates would begin to predominate in the sample.

**Figure 10:**
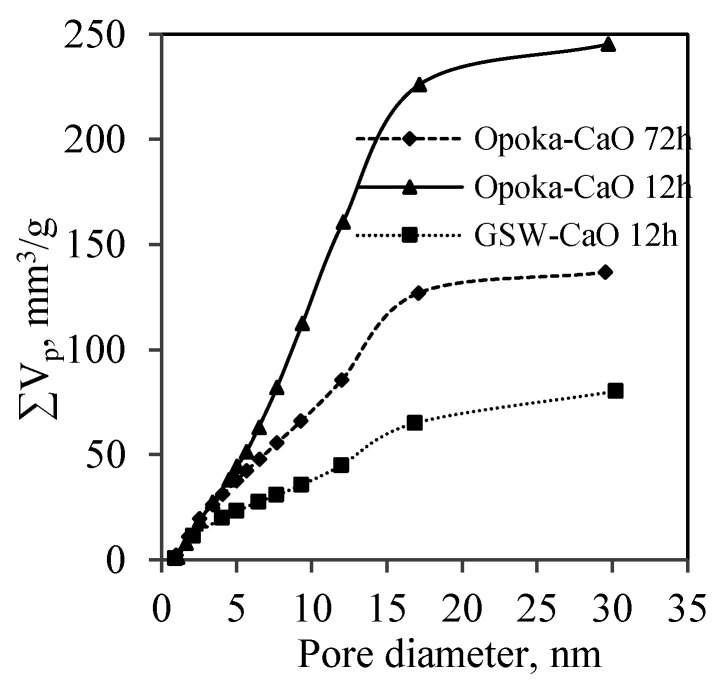
Total pore volume of products (CaO/SiO_2_ = 1.0 at 200 °C) after 12 h and 72 h of synthesis.

It was determined that the highest value of total pore volume (245 mm^3^/g) was obtained in the sample synthesized in the lime–calcined opoka mixture for 12 h (Figure 10). As expected, the total pore volume decreased significantly when the synthesis duration was prolonged up to 72 h, i.e., from 245 mm^3^/g to 137 mm^3^/g (Figure 10). This is in good agreement with the data of *S_BET_* and pore shape calculations (Table 3 and Table 4). Meanwhile, the value of pore volume in the sample obtained from the lime–GSW mixture was equal only to ~80 mm^3^/g.

**Figure 11:**
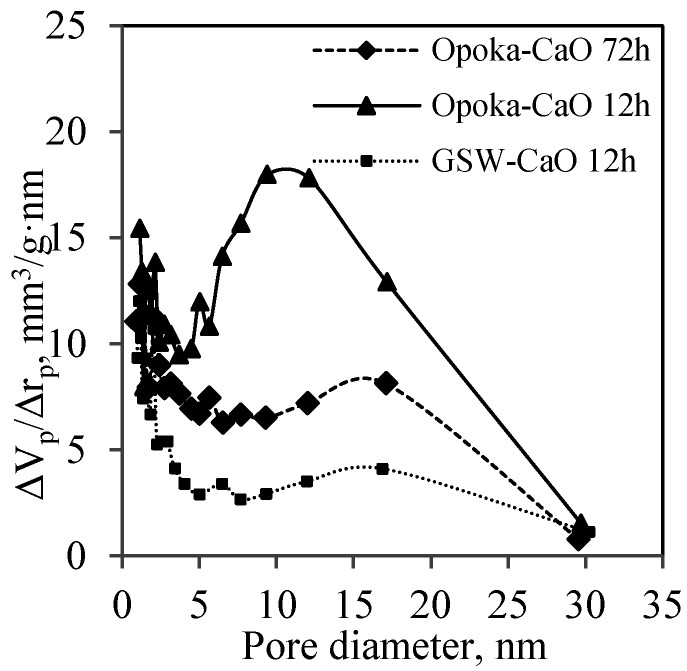
Differential distribution of the pore sizes in the products (CaO/SiO_2_ = 1.0 at 200 °C) after 12 h and 72 h of synthesis.

It was determined that the diameter of pores formed in the synthesis products varied in a wide range, i.e., from 1 nm to 30 nm (Figure 11). It is worth mentioning that the duration and the chemical composition of the primary mixture have an insignificant effect on the diameter of pores. The porosity of the obtained calcium silicate hydrates should provide good thermal insulation properties for the products made from them as no air convection occurs in the fine pores.

## 4. Conclusions

1. It has been found that the granite sawing waste is a suitable material for the synthesis of 1.13 nm tobermorite—it begins to dominate in the product already after 12 h of hydrothermal curing at 200 °C. The size of the crystallites of this compound increases gradually but constantly by prolonging the duration of isothermal curing at 200 °C from 12 h to 72 h. Synthesis of xonotlite from this raw material is not recommended.
2. In lime–calcined opoka suspensions, the formation of crystalline calcium silicate hydrates takes place much faster than in the lime–granite sawing waste mixture. The high reactivity of amorphous SiO_2_ results in the rapid formation of 1.13 nm tobermorite and xonotlite (12 h). After extending the curing duration to 72 h, the size of the crystallites of the formed compounds changes differently. In the case of 1.13 nm tobermorite this value increases only slightly (from 42 to 45.2 nm), while in the case of xonotlite increases by almost 30% (from 22.5 to 28.9 nm).
3. The high reactivity of calcined opoka under hydrothermal conditions is due to its chemical composition, especially the presence of 2.53% Al_2_O_3_ and 0.83% K_2_O. Compounds containing aluminum and potassium ions are evenly distributed throughout the raw material. Al^3+^ ions stimulate the reactions of amorphous SiO_2_ and CaO, which results in the faster formation of 1.13 nm tobermorite in the early stages of hydrothermal synthesis. K^+^ ions accelerate the dissolution of SiO_2_ crystalline modifications (quartz, tridymite, and cristobalite) by destroying the surface of particles and promoting xonotlite formation processes.
4. It was determined that the shape of pores and specific surface area depend on both the chemical composition of the primary mixture and the duration of synthesis. The specific surface area and total pore volume of sample synthesized in granite sawing waste–CaO mixture for 12 h are equal to 25 m^2^/g and 80 mm^3^/g, respectively. The calculations revealed that cylindrical-shape and slit-shape pores are presented in the sample. It was obtained that by extending the duration of hydrothermal synthesis to 72 h, the sample becomes nonporous whose *S_BET_* is equal to ~19 m^2^/g.
5. It was obtained that the cylindrical-shape pores were formed in synthesis products obtained in opoka–CaO mixture. Since a high amount of tobermorite and xonotlite is presented in the samples synthesized for 12 h and 72 h, their specific surface area is quite high and equal to 64 m^2^/g and 40 m^2^/g, respectively. It was calculated that the total pore volume decreased from 245 mm^3^/g to 137 mm^3^/g by prolonging the duration of hydrothermal synthesis. In both samples pores with 1–2.5 nm and 5–20 nm diameter are dominant. Such parameters of synthesis products should provide good thermal insulation properties for the products made from this material as no air convection occurs in the fine pores.
6. The results obtained in the work form the basis in the development of xonotlite-type heat-resistant (up to 1000 °C), low-density (up to 200 kg/m^3^) thermal insulation products from the lime–calcined opoka mixture.

## Figures and Tables

**Table 1:** Chemical composition of raw materials obtained by Rietveld method.

Material	Oxides, wt%								Other	Loss of Ignition, wt%
	SiO_2_	CaO	Al_2_O_3_	K_2_O	Na_2_O	MgO	Fe_2_O_3_	SO_3_		
Opoka	54.60	22.10	2.53	0.83	0.09	0.55	1.66	0.58	0.74	16.41
Granite	58.41	3.95	15.41	3.86	3.45	2.87	7.17	0.19	0.48	4.31
Limestone	4.38	50.88	0.22	0.23	–	1.67	0.70	0.53	–	41.39

**Table 2:** Mineral composition of raw materials.

Material	Minerals						
Opoka	Quartz	Cristobalite	Tridymite	Amorphous Part	Muscovite	Calcite	Dolomite
wt%	8.7	21.1	5.2	19.6	3.9	38.8	2.7
Granite	Quartz	Albite	Anorthite	Labradorite	Microcline	Annite	Actinolite
wt%	23.4	17.8	11.4	13.4	19.2	6.7	7.9

**Table 3:** Calculated parameters of synthesis products obtained from various raw materials.

Raw Materials	Duration, h	BET Equation Constants		C_BET_ Constant	Capacity of Monolayer *X_m_*, g	*S_BET_*, m^2^/g
		Slope *S*	Intercept *I*			
Lime–GSW	12	137.26	2.652 × 10^−1^	52.74	0.0072	24.91
Lime–GSW	72	183.09	2.816 × 10^−1^	66.02	0.0054	18.73
Lime–calcined opoka	12	53.81	6.230 × 10^−2^	87.38	0.0182	63.98
Lime–calcined opoka	72	86.76	1.303 × 10^−1^	67.58	0.0114	39.55

**Table 4:** Values of the measured and calculated specific surface area of the synthesis samples obtained at 200 °C in unstirred suspensions with CaO/SiO_2_ = 1.0.

Mixture	Duration	Calculation Results Using the Cylindrical Pore Model		Calculation Results Using the Parallel Plate Pore Model	
		Σ*A*, m^2^/g	|*S_BET_* − Σ*A*|, %	Σ*A*, m^2^/g	|*S_BET_* − Σ*A*|, %
Lime–GSW	12 h	31.07	24.72	16.73	32.83
Lime–GCW	72 h	-	-	-	-
Lime–calcined opoka	12 h	68.93	7.73	37.82	40.89
Lime–calcined opoka	72 h	50.63	28.01	27.18	31.22

## Data Availability

The data that support the findings of this study are available from the corresponding author, R.S., upon reasonable request.
